# Genes underlying the evolution of tetrapod testes size

**DOI:** 10.1186/s12915-021-01107-z

**Published:** 2021-08-18

**Authors:** Joanna Baker, Andrew Meade, Chris Venditti

**Affiliations:** School of Biological Sciences, University of Reading, Reading, RG6 6BX UK

**Keywords:** Testes size, Genotype-phenotype associations, Evolutionary rates, Phylogenetic comparative methods

## Abstract

**Background:**

Testes vary widely in mass relative to body mass across species, but we know very little about which genes underlie and contribute to such variation. This is partly because evidence for which genes are implicated in testis size variation tends to come from investigations involving just one or a few species. Contemporary comparative phylogenetic methods provide an opportunity to test candidate genes for their role in phenotypic change at a macro-evolutionary scale—across species and over millions of years. Previous attempts to detect genotype-phenotype associations across species have been limited in that they can only detect where genes have driven directional selection (e.g. brain size increase).

**Results:**

Here, we introduce an approach that uses rates of evolutionary change to overcome this limitation to test whether any of twelve candidate genes have driven testis size evolution across tetrapod vertebrates—regardless of directionality. We do this by seeking a relationship between the rates of genetic and phenotypic evolution. Our results reveal five genes (*Alkbh5*, *Dmrtb1*, *Pld6*, *Nlrp3*, *Sp4*) that each have played unique and complex roles in tetrapod testis size diversity. In all five genes, we find strong significant associations between the rate of protein-coding substitutions and the rate of testis size evolution. Such an association has never, to our knowledge, been tested before for any gene or phenotype.

**Conclusions:**

We describe a new approach to tackle one of the most fundamental questions in biology: how do individual genes give rise to biological diversity? The ability to detect genotype-phenotype associations that have acted across species has the potential to build a picture of how natural selection has sculpted phenotypic change over millions of years.

**Supplementary Information:**

The online version contains supplementary material available at 10.1186/s12915-021-01107-z.

## Background

Detecting which genes have driven phenotypic change is a fundamental goal in biology and the subject of many decades of research (e.g. [1, 2]). However, whilst we have a plethora of candidate genes implicated in phenotypes within individual (or a few) species, we still do not know which genes have driven phenotypic change across large taxonomic scales and over millions of years.

Hitherto, approaches for detecting continuous genotype-phenotype associations across species have sought a relationship between the strength of molecular adaptation (i.e. the rate of non-synonymous mutations, dN relative to the rate of synonymous mutations, dS) and the magnitude of a trait of interest, e.g. brain size [3, 4] or plumage coloration [5]. Such an association can reveal where adaptation in a given gene has driven phenotypic change over millions of years—for example, *SEMG2* (encoding a protein involved in semen coagulation) is linked to increasing testes size across primates [6]. However, where no relationship is found using these approaches, it merely indicates a lack of directional change rather than a lack of association per se. For example, *ASPM* (a gene involved in human microcephaly) has been shown using these phylogenetic approaches to drive both brain size increases and decreases across primates [7]. Current approaches are therefore inherently limited by the assumption that individual genes drive only unidirectional change. However, non-homogeneity in evolutionary change is now known to routinely occur at both molecular and phenotypic levels (e.g. [8, 9]).

**Fig. 1:**
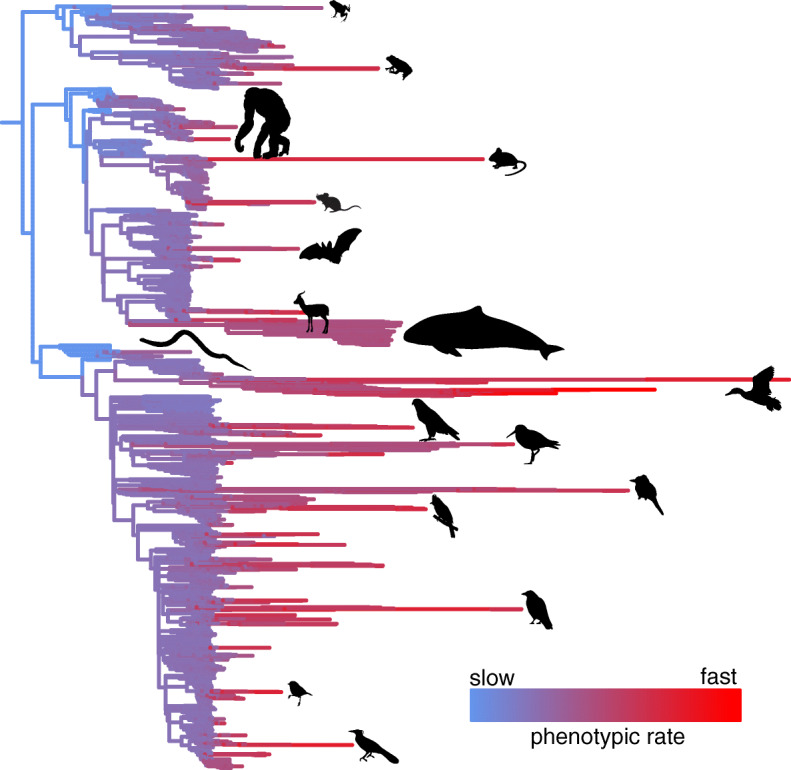
Variation in the rate of testes size evolution across tetrapods. The branches of the tetrapod phylogeny (*N* = 1845) are measured to represent the rate of testes mass evolution (time multiplied by the median of the posterior distribution of rates estimated for each branch, methods). Lineages with comparatively fast rates of evolution in testes mass relative to body mass are represented by longer branches. Branches are additionally coloured according to their relative rate of phenotypic evolution (see scale). Silhouettes are added for purely illustrative purposes to indicate lineages with relatively rapid change in relative testes mass; they are not presented at any scale. All silhouettes are in the public domain and are obtained from phylopic.org

It has recently been revealed that explosive bursts of rapid testes size change occurred during vertebrate evolutionary history [10] (Figure 1). Whilst testes size is a well-studied trait, we still know relatively little about which genes underlie the diversity observed across species. We use the substantial heterogeneity observed in the rate of testes mass evolution (Figure 1) to introduce and apply a novel approach which seeks to detect genes involved in testes size regardless of the directionality of change. Specifically, we test a suite of 12 genes previously implicated in mouse testes mass to determine whether they have played a wider role in the evolution of tetrapod testes size. Our results have the potential to reveal how natural selection at the genetic level can result in significant changes in species’ phenotypes across millions of years.

**Fig. 2:**
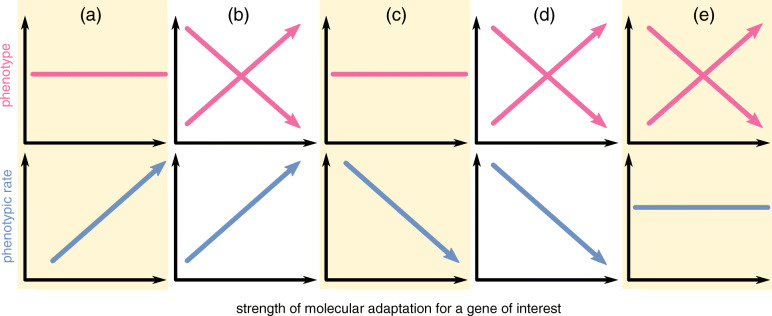
Potential genotype-phenotype links for a hypothetical target gene. The relationship between strength of molecular adaptation (relative rate of protein-coding changes, dN given dS) are shown with magnitude of phenotype in pink and with rate of phenotypic evolution in blue. Crossed lines indicate an identical interpretation regardless of the direction. Where no association is found for the phenotype but is positive for rate (**a**), genetic selection has driven rapid (i.e. adaptive) phenotypic change in both directions during evolution (e.g. ASPM and primate brain size). If there is also an association with phenotype (**b**), interpretation is identical to (**a**), except the adaptation has been consistently directional. Where an association is not found for the phenotype but is negative for rate (**c**), molecular adaptation is explicitly associated with slower rates of phenotypic change—such genes can be thought of as phenotype moderators acting to minimise change. If there is an association also found for the phenotype (**d**), it means that although the gene is acting as a moderator, where molecular adaptation does occur it tends to have been in a consistent direction. Finally, where an association is found only for the phenotype and not the rate (**e**), it implies that this gene has driven directional phenotypic evolution, but phenotypic change has not occurred rapidly and thus may be associated with selection on another, associated phenotype

Here, we introduce an approach to testing for genotype-phenotype associations acting across species and over millions of years that moves away from the limiting assumption of directionality. We harness the power of contemporary phylogenetic comparative approaches that reveal widespread and common heterogeneity in the rate of phenotypic evolution (e.g. [8, 11, 12]) reflecting variation in the strength of natural selection (e.g. [8, 11, 13–17]). What is of critical importance is that this variation in rate reflects periods of phenotypic change in any direction. With this in mind, we seek a relationship between the strength of molecular adaptation and the amount of historical testes size change measured by the relative rate of testes mass evolution (Figure 2).

In the absence of consistent directional phenotypic change, a relationship between molecular and phenotypic rates can reveal a link that would otherwise be hidden by studying only the magnitude of the phenotype (Figure 2a, c). A positive relationship between the rate of phenotypic evolution and the strength of molecular adaptation means that relatively high rates of protein-coding change are directly associated with rapid changes in the phenotype (Figure 2a, b). If protein-coding changes in the target gene have consistently acted to reduce phenotypic change, we would observe a negative relationship between the rate of phenotypic evolution and the strength of molecular adaptation (Figure 2c, d). Such a relationship means that a gene is actively reducing phenotypic variation: it is acting as some sort of phenotypic *moderator* and may have interactions or functional associations with other genes. In both cases, we can also study the phenotype to reveal directionality: where there is also a significant relationship between the phenotype and the strength of molecular adaptation, rapid change has been persistently unidirectional (Figure 2b, d, e.g. *SEMG2*); otherwise, the gene has driven change in both directions (Figure 2a, c). In a situation where we find an association with only the phenotype (Figure 1e), the gene of interest may have driven directional phenotypic change - though not rapidly. 

## Results and discussion

A recent genome-wide association study [18] revealed twelve genes within the mouse genome implicated in variation among testis weight. For each of the 12 target genes (Table 1), we downloaded [19, 20] and aligned [21] orthologous coding sequences for all tetrapods included in the time tree of life [22]. To maximise sample size, we included all available sequences for each gene [see full sequence list in Table S1 in Additional file 1]. We used a maximum-likelihood codon-based substitution model with no site-to-site variation [23–25] implemented in HyPhY [26] to estimate branch-wise dN and dS values independently for each branch of the tetrapod phylogeny [22] (local model).

**Table 1:** Target genes for testis size

Gene	D	df	***N***_**gene**_	***N***_**codons**_	***N***_**Taxa**_	Outgroup
*Alkbh5*	1275.71*	375	193	373	101	*Xenopus*
*Cdkn2c*	582.93*	440	226	166	111	*Latimeria*
*Dmrtb1*	793.65*	388	200	151	97	*Latimeria*
*Dnah11*	5209.60*	311	159	4465	74	*Latimeria*
*Lrp8*	1790.75*	422	217	986	111	*Latimeria*
*Ndc1*	1266.98*	472	243	787	115	*Latimeria*
*Nlrp3*^*a*^	517.70*	232	120	1024	63	*Loxodonta*
*Pld6*	1057.41*	400	205	229	104	*Latimeria*
*Sp4*	1555.50*	464	239	757	117	*Latimeria*
*Spata6*	1179.64*	452	233	488	112	*Latimeria*
*Sp8*	1660.07*	319	163	506	87	*Latimeria*
*Ubb*	534.74*	300	156	126	79	*Xenopus*

**Fig. 3:**
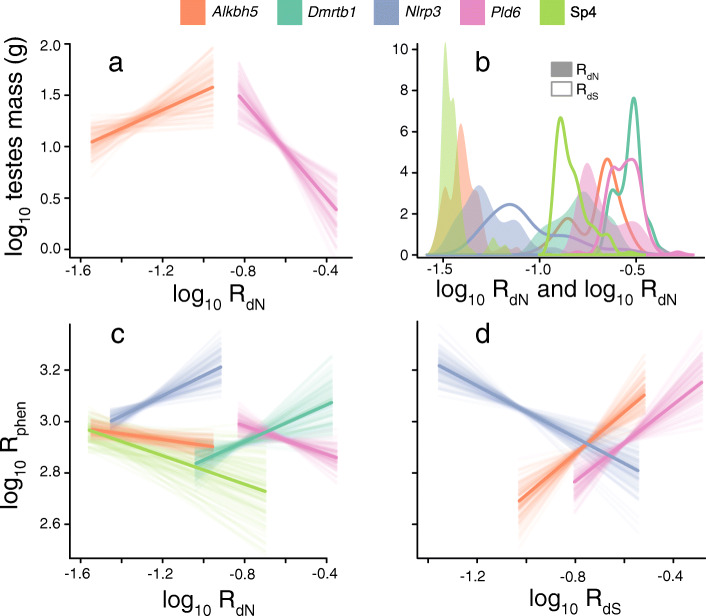
Significant relationships between molecular adaptation and testes mass across tetrapods for five of the twelve studied target genes. The relationship between testes mass and *R*_dN_ (**a**) is significantly positive for Alkbh5 (the proportion of the parameter crossing zero, *p*_x_ = 0.046) and negative for Pld6 (*p*_x_ = 0.002). The relationship between *R*_phen_ and *R*_dN_ (**c**) is significant for all five genes (*p*_x_ = 0.043 for Alkbh5; *p*_x_ = 0.036 for Dmrtb1; *p*_x_ = 0.006 for Nlrp3; *p*_x_ = 0.033 for Pld6; *p*_x_ = 0.048 for Sp4). In both models, *R*_dS_ is included as a covariate, which is always larger than *R*_dN_ (**b**) in line with expectations, e.g. [28]. Whilst R_dS_ is not significantly associated with testes mass for any gene (not shown), it is significantly associated with *R*_phen_ for three genes (**d**): positively in both Alkbh5 (*p*_x_ < 0.001) and Pld6 (*p*_x_ = 0.001) and negatively for Nlrp3 (*p*_x_ = 0.001)

Sample sizes, outgroups, and likelihood ratio (D) test results comparing a global vs. local model of molecular evolution are shown for each gene. *N*_gene_ is the number of taxa included in the model used to calculate dN and dS across the total number of codons (*N*_codons_). *N*_taxa_ is the final number of taxa used in our regression models (Figure 3). *All comparisons are significant at the *p* < 0.001 level . ^a^Note that Nlrp3 is restricted to mammals only—all other genes are analysed across tetrapods

For all twelve target genes, we found significant evidence for branch-to-branch variation in molecular rates of evolution using standard likelihood ratio (*D*) tests (Table 1), comparing the fit of the local model for each gene to one which estimates only a single dN and dS across the whole alignment and phylogeny. To obtain a measure of molecular adaptation, for each species we summed all estimated rates along an evolutionary path of a species, leading from the root to the tip of the phylogeny. We use the root-to-tip dN (henceforth *R*_*dN*_) accounting for the root-to-tip dS, *R*_*dS*_ (i.e. in Bayesian phylogenetic multiple regression analyses) as our measure of variation in the strength of molecular selection (Figure 2)—this allows for the potential that dS may be affected by fluctuating environments or ecological variability [27, 28].

We followed the approaches described in Baker et al. [10] in order to measure the rate of relative testes mass evolution across the branches of the tetrapod tree of life [22]. We used the variable rates model to measure rate heterogeneity in the phylogenetically structured residual error of the regression relationship between testes mass and body mass (n = 2036 vertebrate species, see Table S2 in Additional file 1 for data and references). The variable rates model simultaneously estimates three components [8, 11]: (i) the parameters of the regression model; (ii) an underlying background Brownian motion rate of evolutionary change (σ^2^_b_); and (iiii) a set of rates, *r*, which define whether each branch is evolving faster (*r* > 1) or slower (*r* < 1) than σ^2^_b_. We multiplied the original branch lengths of the phylogeny (measured in millions of years) by *r* to give a rate of testes size evolution along each individual branch relative to time (*rt*). Using Log Bayes Factors [29] (BF, see the “Methods” section), we found significant heterogeneity in the rate of testes size evolution across tetrapods (Figure 1): BF = 959.41 compared to a model estimating only a single rate of evolution. As with the molecular rates, we calculated the sum of all branch-wise *rt* for each species from root to tip as a measure of the total changes in phenotypic rate a species has experienced during its evolution, *R*_*phen*_.

Our analyses provided three components of historical adaptation that have acted along the branches of the tetrapod phylogeny: (i) *R*_*dN*_, (ii) *R*_*dS*_, (iii) and *R*_*phen*_. We used these components to test whether molecular adaptation (i.e. *R*_*dN*_ accounting for *R*_*dS*_) in each of the twelve target genes has been linked to evolutionary change in testes mass. We conducted two sets of phylogenetic generalised least squares regression models implemented within a Bayesian framework [8], testing for an association between *R*_*dN*_ and either: (i) testes mass or (ii) *R*_*phen*_. In all models, we include both *R*_*dS*_ and body mass as covariates; for the second set of models where *R*_*phen*_ is the response, we additionally include testes mass as another covariate.

We find significant associations in five of the twelve target genes (Figure 3). However, just two of the target genes have a directional association between *R*_*dN*_ and testes mass (after accounting for *R*_*dS*_): natural selection has driven testes size increase via *Alkbh5* and decrease via *Pld6*. One of these genes (*Alkbh5*) has been previously heralded as a “*top candidate gene*” for testes size [18] and both genes have been demonstrated to directly affect testes mass using knock-out experiments [30–32]. On the other hand, all five of the genes in which we find significant associations demonstrate a significant relationship between *R*_*dN*_ and *R*_*phen*_ (Figure 3, after accounting for *R*_*dS*,_ testes mass and body mass). This highlights the ability of our approach to overcome the limiting assumption of directionality [33], making it possible to find genotype-phenotype associations in the absence of any unidirectional relationship (e.g. *Nlrp3*, Dmrtb1 and *Sp4*, Figure 3a,c).

Three genes (*Alkbh5*, *Pld6*, *Sp4*) appear to be moderators of testes size (Figure 3c), i.e. they demonstrate a significant negative association between *R*_*dN*_ and *R*_*phen*_ (as in Figure 1c,d). This is the only association found for *Sp4* (Figure 3), indicating that this gene is a simple testes mass moderator (i.e. Figure 2c) acting to minimise evolutionary changes in testes mass across tetrapods. However, both *Alkbh5* and *Pld6* appear to act as moderators with specific directionality (e.g. Figure 2d). Both genes show a general link between testes size change and the mutation rate (a significant positive association between *R*_*phen*_ and *R*_*dS*_, Figure 3d), but where there has been strong molecular adaptation (higher relative *R*_*dN*_), testes size change is minimised (lower *R*_*phen*_, Figure 3c). Where protein-coding changes do result in testes size change, these tend to be towards larger (for *Alkbh5*) or smaller (for *Pld6*) size (Figure 3a). The three genes for which we detect a moderator role are each known to act to affect the behaviour or function of other proteins. *Sp4* is a transcription factor and by its very nature involved in transcription. *Alkbh5* is an RNA demethylase that acts to moderate and remove m6a base modifications [31]. High levels of m6a modifications are implicated in male infertility [30], and they are also thought to play a key role in spermatogenesis [34]. *Pld6* is involved in piRNA biogenesis [32]—an essential molecule involved in gene silencing [35] that also has demonstrated links to spermatogenesis [36]. Our results imply that the three moderator genes we reveal here may have functional relationships with other genes acting on tetrapod testes sizes whilst also (for *Alkbh5* and *Pld6*) demonstrating a clear directional effect on testes themselves [30, 31] (Figure 3a).

In two genes, *Nlrp3* and *Dmrtb1*, protein-coding changes have driven increased testes size variability, but they have not driven directional change throughout tetrapod evolution (Figure 3a, c). That is, increased levels of protein-coding change are associated with increased levels of testes size change. Although little is known about the specific function of *Dmrtb1*, it has been linked with murine spermatogenesis and is co-expressed with DMRT1 [37, 38], a gene that is fundamental to male sex determination and that must be expressed throughout an individual’s life to maintain sexual identity [39, 40]. Missense mutations in *Nlrp3*, a gene dominantly associated with innate immune system function [41], have been linked to altered fertility in mice [18, 42]. Elevated rates of molecular change in immunity genes in primates have previously been linked to promiscuity and the potentially increased risk of sexually transmitted diseases [43].

In accordance with the nearly neutral theory of molecular evolution [28, 44], all twelve genes had much faster rates of synonymous substitutions compared to non-synonymous rates (Figure 3b, both are calculated relative to each other, see the “Methods” section). There is also an expected ubiquitous strong significant correlation between both *R*_*dN*_ and *R*_*dS*_ as well as branch-wise dN and branch-wise dS. However, in *Nlrp3*, we find a negative relationship between *R*_*dS*_ and *R*_*phen*_ (Figure 3d). That is, after accounting for *R*_*dN*_ (and both body mass and testes mass), synonymous mutations have acted to reduce rapid evolutionary changes in testes mass. This unexpected negative association implies that, for this gene at least, synonymous substitutions are likely to not be silent to selection. This is in line with evidence that not only can the dN/dS ratio vary substantially across loci [45], but also that synonymous mutations can explicitly alter the expression and function of the translated protein [46, 47]. Indeed, elevated *Nlrp3* expression has recently been detected in testes tissue compared with that of other organs for both rodents and primates [48].

Of the twelve genes we studied here, there were seven genes in which we found no significant relationship between the rate of molecular evolution and the evolution of testes mass across tetrapods (*cdkn2c*, *Dnah11*, *Lrp8*, *Ndc1*, *Sp8*, *Spata6*, *Ubb*). However, we do not interpret this to mean that these genes are not necessarily important in the evolution of tetrapod testes size. For example, they may still be implicated in the evolution of other phenotypic characteristics such as sperm morphology, Sertoli cell development, and sperm motility. For example, *Spata6* has been shown to be associated with “*pinhead sperm*” (a morphological abnormality wherein spermatozoa have no or very small heads) [49]; variability in phenotypes such as sperm morphology may not be reflected well by gross measures of testes morphology such as mass [50]. Increasing availability of phenotypic data may allow future studies to tease apart more nuanced roles for all the genes we study here—and many beyond.

The twelve target genes that we study here are not the only ones that have previously been implicated in testes size variation (e.g. [6]). We also know that there have been different patterns of testes size change in different vertebrate groups [10] and for species with varying intensities of sperm competition [51]. Using our approach alongside the increasing availability of molecular data, it is now possible to begin to tease apart which genes have played a role in testes size evolution as well as understanding their underlying ecological drivers.

## Conclusions

We introduce and implement a novel approach to detecting genotype-phenotype associations in order to reveal five genes that are likely to have played a key role in driving the diversity in testes size across tetrapod vertebrates. Our approach demonstrates a way to detect which genes might have driven evolutionary change in the absence of directional selection. This provides a novel and exciting opportunity for detecting previously hidden gene-phenotype associations across long evolutionary scales. In the future, it should be possible to use this approach to screen suites of genes including potential whole-genome scans for impacts on relevant morphology, allowing us to build the picture of how natural selection has sculpted diversity—from genes to protein to phenotype over millions of years of evolutionary history.

## Methods

### Data

The phylogenetic tree used for all of our described analyses (molecular, phenotypic, and combined) was the vertebrate time tree of life [22] (downloaded January 2020 using the TimeTree web resource, http://www.timetree.org/). In each analysis, we limited the phylogeny to only include taxa with data (see below for more details, and also Tables S1-S3 in Additional File 1).

In a recent genome-wide association study, Yuan et al. [18] identified three quantitative trait loci (QTL) within the mouse genome (*n* = 502) that are associated with variation among testis weight [18]. Within these three regions, a total of 36 genes were found to have previously been implicated in reproductive phenotypes, but only a subset of these were found to have experienced natural selection (dN/dS ratio > 1). Whilst it is possible to reveal associations using our approach in the absence of positive selection (Figure 2), genes with known positive selection are more likely to have experienced significant heterogeneity in dN and dS during tetrapod evolution. We therefore identify 12 target genes from the analyses presented in Yuan et al. [18] to which we apply our approach to detect whether any have played a key role in the evolution of tetrapod testis size (Table 1).

For each of the twelve genes named in Yuan et al. [18], we downloaded orthologous sequences using the NCBI orthologue browser [19] and Ensembl [20], using mouse sequences as reference. We only included one-to-one orthologues (excluding species with multiple copies of the gene). Spurious sequences were identified both by visual assessment and automatically using trimAL [52], retaining only sequences where at least 80% of all sites overlap with at least 75% of sequences in the alignment. We also downloaded an outgroup sequence for each gene: the coelacanth was preferred, but where this was not available, we used an alternative suitable outgroup (Table 1). After removal of spurious sequences, multiple alignments were generated using MUSCLE [21]. Each multiple alignment was then matched to the time tree of life [22] and uninformative sites were removed using trimAL’s [52] heuristic algorithm optimised for trimming alignments analysed by maximum likelihood phylogenetic analyses. A full list of accession numbers for all sequences used in our analyses can be found in Table S1 [see Additional file 1]. The sample size for each gene can be found in Table 1.

Testes mass (g) and body mass (g) data was obtained from Baker et al. [10] and updated to include 2036 species matched to the updated time tree of life [22]. We provide the full testes size dataset and reference list in Table S2 [see Additional file 1]. The final dataset comprised testes mass and body mass measurements for 992 birds, 621 mammals, 200 amphibians, 32 squamates and 191 fish. Taking a wider taxonomic perspective to estimate the phenotypic rate (i.e. across all vertebrates rather than across tetrapods) allowed us to more accurately estimate the background rate of evolution [8], but all subsequent analyses were performed at tetrapod level owing to genome duplications in teleost fishes [53]. All data was log_10_-transformed before analysis.

For both genetic and phenotypic data, we only include species that are included in the tetrapod time tree of life. In some cases, species were included by genus matching (see Tables S1-S3 for details, all included in Additional file 1). However, our results do not differ if these species were excluded.

### Phenotypic rate variation

Variation in the rate of testes mass evolution after accounting for body size was detected using the *variable rates regression model* [8, 11] following Baker et al. [10] and using the time tree of life [22] with branch lengths measured in millions of years. We used a regression model that estimated a separate relationship between testes mass and body mass for each of the five major clades studied (fish, frogs, birds, mammals and reptiles) in line with previously published results [10]. The variable rates regression model works within a phylogenetic Bayesian Markov Monte Carlo (MCMC) framework to estimate rate heterogeneity in the phylogenetically structured residual error of our regression model along the branches of the tree [8] *.* For each branch, the model estimates a posterior distribution of rate scalars *r* which define whether each branch is evolving faster (*r* > 1) or slower (*r* < 1) than the overall background rate of testes size evolution—which is estimated simultaneously as an underlying Brownian motion rate (σ^2^_b_). We then stretch (or compress) the original branch lengths of the phylogeny measured in millions of years by the median branch-specific *r* values to create a *phenotypic rate-scaled* phylogenetic tree where branches reflect the inferred rates of evolutionary change in testes size during the course of vertebrate evolutionary history. In this rate-scaled phylogeny, stretched branches reflect increased rates of morphological evolutionary change whereas compressed branches represent lineages where testes mass has changed less than would be expected given σ^2^_b_. We ran each model for a total 1 billion iterations after convergence, sampling every 500,000. All chains were run 5 times and checked visually to ensure convergence. Results are qualitatively identical across all replicates.

The presence of significant heterogeneity in the rate of phenotypic evolution was determined using a Log Bayes Factor (BF = -2log_e_[m_1_/m_0_])—where m_1_ is the marginal likelihood of our variable rates model and m_0_ is the marginal likelihood of a model with a single underlying background rate, σ^2^_b_) assessed against the table provided by Raftery [29]. Marginal likelihoods were estimated in BayesTraits v3.0.1 (www.evolution.rdg.ac.uk/BayesTraitsV3.0.1) using a stepping-stone sampler [54] where we sampled 1 million iterations for each of 500 stones, drawing values from a beta-distribution (*α* = 0.40, *β* = 1) [54]. Where we found significant phenotypic evolutionary rate heterogeneity, we obtained species-specific values of phenotypic rate variation. To do this, we created a phenotypic-rate scaled phylogeny using the median set of *r* values estimated in our variable rates model. We then summed all rate-scaled branches along the evolutionary path of each species from root to tip. This is a measure of the total amount of change in phenotypic rate a species has undergone during its evolutionary history [13], termed here *R*_*phen*_. Any non-independence in *R*_*phen*_ among taxa is directly accounted for by using a phylogenetic comparative model which explicitly incorporates shared phylogenetic history (i.e. shared path lengths). Note that our path lengths are measured from the phylogenetic tree that is scaled using the median rate scalar acting along each branch. Whilst the median represents a suitable summary value, future advancements may allow us to incorporate the full posterior distribution of path lengths and rates acting throughout the phylogeny.

### Molecular rate variation

Variation in the rate of molecular evolution was detected using codon-based substitution models as implemented in HyPhy [26]. All analyses were run on the time tree of life, removing species for which we did not have sequence data (see Table S1). The tree was then unrooted and branch-length information (in millions of years) was removed. For each gene, we ran two MG94xREV [24, 55] codon models to estimate separate independent synonymous (dS) and non-synonymous (dN) rates of evolution [56], but did not allow site-to-site variation. The first was a local model, where a single dN and dS was estimated across the full alignment and the second was a global model where a separate dN and dS was estimated for each individual branch. We assessed whether there was significant evidence for branch-to-branch variation in molecular rates of evolution using standard likelihood ratio (*D*) tests, comparing the fit of the local and global models.

Where we found significant evidence for branch-to-branch variation in dN and dS (likelihood ratio tests, see below), we obtained species-specific values of molecular rate variation in the same way as described for phenotypic rate variation above. We created two new trees, where branch lengths are respectively measured as the branch-specific dN and dS as estimated by our codon models. These trees were rooted on the outgroup taxa which was then removed. The branch lengths of these molecular rate trees are proportional to the instantaneous rate and are therefore time-independent. However, time is accounted for in all our models both as a part of the phylogenetic tree (where branches are measured in time) and within the phenotypic rate (see above for more details). We then summed all branches from root to tip for each species to obtain R_dN_ and R_dS_: measures of the total amount of change in dN and dS a species has experienced during its evolution. We did this independently for each gene. As with *R*_*phen*_, non-independence in R_dN_ and R_dS_ is directly accounted for by using a phylogenetic comparative model (see below). Using these root-to-tip measures and accounting for the shared evolutionary history among them allows us to study substitution rates (and phenotypic rates) that are much more in line with taxon-level properties (testes size, body size, etc.) than by using alternative approaches such as by studying terminal branches alone [57]. Studying the rate of evolution (be it molecular or phenotypic) throughout the whole tree can also give increased statistical power [57].

### Detecting genotype-phenotype associations

We used our data on testes mass, body mass, and phenotypic and molecular rate heterogeneity in order to determine whether any of the proposed candidate genes [18] have driven changes in testes mass during the course of tetrapod evolutionary history.

We determine whether each gene has had any directional effect on testes mass evolution using a PGLS regression model estimating the relationship between testes mass, *R*_dN_ and *R*_dS_—whilst also accounting for body size. Sample sizes were limited to those species which had both molecular and phenotypic data (see Table 1) and were not large enough to test this relationship independently in different taxonomic groups. We then determined whether any changes in selection pressure on each individual gene have led to any adaptive response in testes mass by conducting a second PGLS regression in which we test the effects of testes mass, body mass, *R*_dn_ and *R*_dS_ on *R*_phen_. All data were log_10_-transformed before analysis.

All PGLS models are conducted in a Bayesian MCMC framework using BayesTraits and estimate lambda [58] as a measure of phylogenetic signal. All models were run on the time tree of life including only those species for which we had both molecular and phenotypic data. All chains are run for 1 million iterations, sampling every 500 thousand iterations after convergence. We assess parameter significance by calculating the proportion of the posterior distribution of a parameter estimate that crosses zero (*p*_*x*_)—where *p*_x_ < 0.05, less than 5% of the distribution crosses zero and we consider the parameter to be significant.

Our approach is uniquely placed to detect genotype-phenotype associations that have acted across species. A recently proposed method, *RERconverge* [59] seeks to link trait change to rates of molecular evolution, but differs from our approach in several important ways: firstly, our approach allows us to control for multiple explanatory factors. Here, we account for both testes mass and body mass to rule out any spurious associations owing to a possible general association between the rate of molecular evolution and testes size and body size. Secondly, *RERconverge* relies on non-specific rates of genetic evolution that do not distinguish between non-synonymous and synonymous changes. Finally, *RERconverge* depends on ancestral state reconstruction to estimate phenotypic rate heterogeneity.

### Ensuring robustness of results

Following the rationale and logic of Montgomery et al. [4], we conducted additional analyses to determine the specificity of the associations we find and exclude the possibility of type 1 errors. We repeated our main analyses (detecting molecular rate variation and genotype-phenotype associations) on nine additional loci identified from human orthologues with no known implications for testes size (*PAX6*, *MC1R*, *MAOA*, *FOXP2*, *ENAM*, *GATA2*, *MATK*, *SHH*, *TYRP1*—see table S3 [Additional file 1] for all accessions). This control set includes genes that have been previously demonstrated to be under positive selection among various vertebrate lineages and had sequences available for a large subset of taxa overlapping with the testes size data. In line with this, we find evidence for molecular rate heterogeneity (*p* < 0.001, see table S4 in Additional file 1) for all nine control genes. However, when we test for an association between testes mass or the rate of testes mass evolution and the rate of molecular evolution, we find no evidence for any significant relationship. That is, we found no additional loci with significant implications for the evolution of tetrapod testes. This is good evidence that the significant associations we find in our candidate genes represent real biological effects and are unlikely to be type I errors.

For each of our regression models, we excluded each species one-by-one (for each gene we therefore a number of models equal to the number of species included in the full model, i.e. *N*_taxa_ in Table 1) to ensure no individual values for any of the measured data were driving the relationships (or lack thereof) we detected. Our results are robust to this process: we find identical results regardless of which species is excluded. We also repeated the analyses excluding amphibians: a general paucity of sequence data for amphibians means that for most genes we study here, there are only one or two sequences available (Tables S2, S3 in Additional File 1). We find no differences in the results: it seems that amphibians are not unusual with regards to the genes involved in tetrapod testes mass evolution. However, as more genomic data for amphibians becomes available, future investigations could verify this.

For the twelve target genes, several orthologues are classified as “low-confidence” orthologues by ENSEMBL [20] (e.g. *ENSANIP00000026038*, see Table S1 in Additional file 1), meaning that they are below the threshold of one of the three following criteria: (i) Whole Genome Alignment scores which measure how well orthologous genes fall within aligned genomic regions, (ii) a gene order conservation score measures how much an orthologue falls within a block of genes and in the same order amongst its closest relatives, and (iii) and the percentage identity compared with the original sequence (in this case, the mouse). Note that the values of these thresholds vary depending on the taxa being studied; see ENSEMBL help pages [20]. Our results remained completely unchanged when we repeated our analyses excluding all species that are low-confidence orthologues from the calculation of molecular rates in HyPhy—including all post-hoc regression models. Our results are also unaffected if we exclude “low-quality” sequences from GenBank [19], i.e. those that have been altered to correct for potential mismatches between the nucleotide and protein sequences (e.g. Accession *XM_003732882.3*, see Table S1 in Additional file 1).

## Supplementary Information

**Additional file 1.**

## Data Availability

All sequence data is available as described in the “Methods” of this paper. We provide all accessions and IDs for all sequence data used in this study in Additional File 1 (Tables S1, S3). Testes mass (g) and body mass (g) data was obtained from Baker et al. [10] and updated to include 2036 species matched to the updated time tree of life [22]. This updated dataset is also included within Additional File 1 (Table S2).
